# ‘It Is Good to See the Person As a Whole Person and… Continue to Improve Our Psychologically Informed Working’: A Thematic Analysis of Clinical Psychology Trainee Placements in Homelessness Settings

**DOI:** 10.1111/hex.14121

**Published:** 2024-06-23

**Authors:** Rebecca J. Ward, Frances T. Greenway, Nick Maguire

**Affiliations:** ^1^ Centre for Homelessness Research and Practice University of Southampton Southampton UK

**Keywords:** ‘clinical psychology’, homelessness, placements

## Abstract

**Objectives:**

The National Framework for Inclusion Health identified the need for collaborative action between the NHS and third sector health to improve access and outcomes for Inclusion Health groups. Clinical psychology trainee placements in homelessness settings could be a valuable pathway to improving access to psychological support for people experiencing homelessness and the provision of clinical services, which is key to developing the workforce and a catalyst for the future recruitment of clinical psychologists in the third sector.

**Methods:**

A qualitative evaluation was conducted using semistructured interviews to explore the perspectives of clinical psychology trainees, supervisors, staff in homelessness settings and a peer mentor. Twenty‐two participants were recruited from two universities and six services across the South East, including 11 clinical psychology trainees, six supervisors, four placement staff and one peer mentor.

**Results:**

Placement staff described the value of a psychological approach but identified some challenges to be overcome. Induction was identified as the key to success. Supervisors recognised the breadth and depth added to trainees' knowledge and skills alongside significant challenges. Trainees valued the opportunities to work in homelessness settings and develop their understanding of the role. The peer mentor identified collaborative working as especially important.

**Conclusions:**

Clinical psychology trainee placements are a necessary programme to fulfil the NHS vision for Inclusion Health. These placements equip the health and social care workforce to create excellent and sustainable provisions to improve the physical and mental health of people experiencing homelessness whilst also providing much‐needed psychological support for staff.

**Patient and Public Contribution:**

- **Psychologically Informed Environments Through Staff Training:** Staff training and support within these placements contribute to the development of psychologically informed environments. This not only leads to better outcomes for both staff and clients but also aligns with the objectives of the National Framework for Inclusion Health, fostering sustainable provision for the health needs of people experiencing homelessness (PEH).
- **Enhanced Therapeutic Adaptability:** Trainees gain invaluable experience in adapting therapy to meet the diverse needs of clients, benefiting both trainees and clients alike. This adaptability fosters more effective therapeutic relationships and contributes to the improvement of inclusion health provision in the long term.
- **Tailored Therapy for Timely Intervention:** Clinical psychology trainee placements in homelessness settings offer therapy that bypasses long waiting times for interventions, crucial for individuals experiencing homelessness. This flexible approach caters to the unpredictable engagement levels of PEH, ensuring timely support aligning with the Health and Care Act 2022 to improve overall health and address health disparities through primary care networks.

## 1 Introduction

Health inequalities describe unfair, avoidable and systematic differences in people's health and healthcare [1]. These inequalities are driven by the availability and access to care, the quality and experience of care and the varied opportunities for living a healthy life, influenced by wider determinants of health, such as poverty and housing quality [1]. One approach to tackling health inequalities has been the Inclusion Health Framework for those ‘socially excluded, who typically experience multiple interacting risk factors for poor health, such as stigma, discrimination, poverty, violence and complex trauma’ [2].

The Health and Care Act 2022 [3] (Section 14Z35) identified the reduction of inequalities in accessing health services as a legal responsibility for NHS services. The NHS Long‐Term Plan aims to address health disparities through primary care networks [4]. Consequently, NHS England has produced the National Framework for Inclusion Health, with principles for practical action and collaboration between the NHS and third sector health, including developing the workforce to improve access and outcomes for inclusion health groups, such as people experiencing homelessness (PEH; [2]).

Clinical psychologists provide psychological treatment within the NHS to address mental health issues. However, mental health pressures in England have resulted in long waiting lists, funding issues and a workforce too small to cope with demand [5]. Additionally, people in the highest levels of deprivation are least likely to access mental health services [6]. The Doctorate in Clinical Psychology (DClinPsych) programme, funded by Health Education England, employs trainees through NHS Foundation Trusts. Trainees complete supervised practical placements to qualify as clinical psychologists [7]. Typically, placements are closely intertwined with the academic curriculum, serving as a platform for students to translate theoretical knowledge into real‐world practice [8]. Placements traditionally take place in statutory services, but placements within third sector homelessness services can enhance the learning and professional experience of trainee clinical psychologists, as well as provide homelessness staff with a greater understanding of psychologically informed environments; a key approach to shaping services in a psychological way [9]. This can enhance protective and supportive factors encouraging better staff outcomes and ultimately client outcomes.

Principle 3 of the National Framework details developing the workforce for inclusion health [2]. The NHS has provided funding for a pilot of DClinPsych placements in the third sector, specifically in homelessness services. For PEH, this creates the opportunity to receive psychological support in familiar settings such as hostels and day centres and reduce waiting times as trainees become additional members of the workforce delivering interventions and support. Trainees are also able to provide training and support for frontline staff, addressing stress and burnout. However, as third sector placements are not configured to the DClinPsych programme, it may be more challenging to meet the course requirements, placing additional stressors on trainees, supervisors and staff.

There are multiple benefits to trainee placements in healthcare settings when serving PEH. First, trainees experience a broader range of patient care early in their careers, developing an understanding of different populations and how to adapt treatments for a range of patients. Second, services benefit as trainees share their learning and contribute to the adaptation of services to better serve this vulnerable population [10]. Third, trainees can offer support and training to staff, such as reflective practice. Reflective practice enables professionals to gain new insights and recognise complex issues encountered within their organisations or own practice by exchanging ideas and knowledge with colleagues [11], potentially contributing to a reduction in stress, burnout and subsequent turnover [12]. Fourth, these placements can improve skills and knowledge as well as reduce fear and anxiety about working in this setting [13]. To date, there is no published research on clinical psychology trainee placements in homelessness services, although research on placements for other trainee health professionals has demonstrated improvements in trainee understanding of homelessness and the unique needs of this population [14, 15].

Trainee DClinPsych placements in homelessness settings could be a valuable pathway to improving access to psychological support for PEH and the provision of clinical services, key to developing the workforce both in terms of immediate delivery of training and support and a catalyst for future recruitment of clinical psychologists in the third sector. This qualitative evaluation is important in assessing the practicality, usefulness and value of these placements. It is the first study to explore clinical psychology trainee placements in homelessness settings from the perspectives of staff in homelessness services, supervisors of clinical psychology trainees, trainees and a peer mentor.

## 2 Methods

The study was designed in collaboration with an assistant psychologist, DClinPsych programme lead and an expert by experience. The expert by experience emphasised the importance of seeking diverse perspectives and exploring how the findings could enhance services for individuals facing homelessness. They recommended using semistructured interviews to mitigate hierarchical structures and group dynamics between supervisors, trainees and staff, ensuring that participants did not feel pressured to conform to their colleagues' opinions [16]. Twenty‐two participants were recruited from two universities and six services across the South East, including 11 clinical psychology trainees (six first‐years, four second‐years and one third‐year) who had undertaken a placement in homelessness services; six academic or clinical supervisors who had supervised trainees undertaking homelessness placements; four placement staff; and one peer mentor.

Data were collected between October 2022 and September 2023. Individual interviews were conducted to understand the range of views and perspectives on placements in homelessness. A semistructured topic guide covered learning from placements, comparisons with other clinical placements, improvements to homelessness service placements and outcomes from homeless service placements. Interviewees were explicitly given the opportunity to share any further views.

DClinPsych programme managers were asked permission to advertise the study to their student cohort. Similarly, homelessness service managers were approached and invited to advertise the study to their staff. Information packs were sent to those who expressed interest, including the consent form and a short questionnaire collecting information about placement or organisation. All interviews were conducted via Teams, video‐recorded and transcribed verbatim. Interviews lasted 20−68 min (average 40 min).

### 2.1 Ethical Considerations

Ethical approval was granted by the University of Southampton School of Psychology Research Ethics Committee (ERGO ID: 78201). Interested participants were required to provide informed consent before being interviewed. Once transcription and anonymisation were complete, video files were deleted. Digital files were stored on password‐protected computers only accessible by the research team. Confidentiality was assured and participants were informed that only anonymised quotes would be used so that neither the participant nor the participating university could be identified.

### 2.2 Analysis

Reflexive thematic analysis was used in an iterative, cyclical process across the data set [17, 18]. Staff, supervisor, trainee and peer mentor interviews were group analysed to generate group themes using Taguette online software. A critical realist approach was taken in which the perspectives, views and experiences of participants were accepted as real and meaningful [19, 20].

Once transcripts were finalised, familiarisation with the data enabled initial ideas and areas of importance to be identified and sections of text were tagged with a meaningful description or code. Related codes were then brought together to produce themes. Thematic maps on MindMup were generated throughout the analysis and used to develop the final themes.

## 3 Results

Themes are presented within the four perspectives of staff, supervisors, trainees and a peer mentor.

### 3.1 Staff Perspective (See Figure 1)

**Figure 1:**
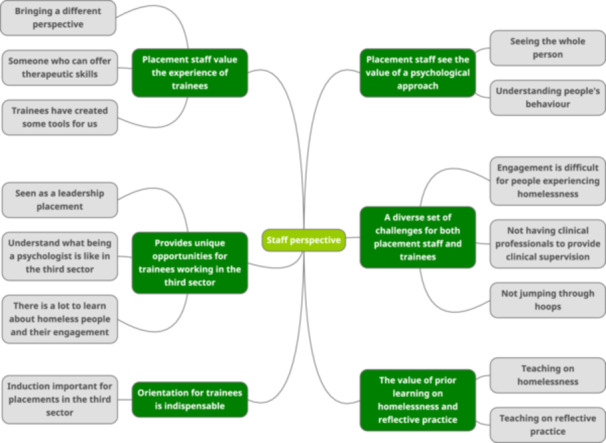
Thematic map for staff perspective.

Figure 1 shows the themes and subthemes resulting from the reflexive thematic analysis of staff interviews.

#### 3.1.1 Placement Staff See the Value of a Psychological Approach

Staff described the value of the psychological approach as seeing the whole person and understanding people's behaviour, rather than focussing on one aspect of their life, one difficulty or issue that needed resolving. It changed their perceptions from seeing negative behaviour as an attempt to antagonise or annoy them to an understandable response to their current experience based on past trauma.

It is good to see the person as a whole person and the trauma that has caused the behaviour…we're really, really keen to continue to improve our psychologically informed working.

[01]

#### 3.1.2 Placement Staff Value the Experience of Trainees

Staff recognised the different expertise and perspectives that trainees brought to the service. Staff training and support is one of the five key areas of psychologically informed environments, but training can be expensive and difficult to organise in services [9]. By having trainees directly in the service, staff also benefitted from training in which trainees shared ideas and information that would equip them in their support of clients.

The main thing is like…having a different perspective and being another person that we can get some ideas from about how to work with some of our more tricky clients. 

[15]

Trainees were able to offer psychological therapy to clients, which was normally offered through external services with long waiting lists and so the trainee placement enabled therapy to be brought into the service and reduce waiting times. Compared to qualified clinical psychologists, trainees are less constrained by caseloads, able to work more flexibly (including across services) and can contribute where needed.

This was something new…to have a student who could offer therapeutic interventions to our young people who can be waiting a couple of years for that kind of instruction.

[01]

#### 3.1.3 A Diverse Set of Challenges for Both Staff and Trainees

Trainees use placements to build experience and evidence competencies for their clinical programme. Engagement is difficult for PEH, who do not always find it easy to keep appointments or follow a therapy plan and, when appointments are missed, trainees may interpret missed appointments as a reflection on their competence and staff were keen to help trainees understand that missing appointments was common.

I think confidence building is one of the things that I think they need to work on…be less self‐critical after interactions and take it less personally. 

[15]

In some services, such as day centres, clients accessing the provision are given the freedom and flexibility to engage in their own way. Services were not willing to jump ‘through any hoops’ [15] to provide a caseload to trainees. This is a different approach to, for example, a mental health team where a trainee may be given a caseload and plan therapy sessions to meet their training requirements.

Another challenge for homelessness services that would like to offer a placement was a lack of internal clinical professionals to provide supervision to trainees. However, one solution was to partner with another service who were able to provide that supervision so the community service could still benefit from a placement.

We didn't think that we would be able to do that on our own. So we said no and then we had an approach from via the mental health homeless team to share…because I don't have that level of expertise or knowledge to manage someone in a clinical way. 

[15]

#### 3.1.4 Provides Unique Opportunities for Trainees Working in the Third Sector

Staff felt that trainees could learn so much from PEH and the services provided for them, but recognised how different the placement experience can be from other services. Engagement was an issue identified as key learning in this sector given that, in other services, trainees may have experienced high engagement from clients. However, staff felt strongly that there was a lot to learn about engagement.

My top tip. Don't panic. People do engage, they will engage. It's just, I think everybody has an idea of counselling [where]…if you're paying £60, £70 an hour you will turn up because you don't want to waste your money. But I think there is a lot to learn about homeless people and their engagement. 

[01]

Understanding what being a psychologist is like in the third sector, including developing your identity as a psychologist, was identified as important as there are many aspects to what a clinical psychologist can do: including using a variety of psychological models in therapy; understanding the wider societal and community issues relating to homelessness; and building productive working relationships with other services. Therefore, experiencing different sectors was a positive outcome of these placements.

Some staff considered the placement to be beneficial for trainees in their third year rather than near the beginning of their training, as it was seen as a leadership placement. This was largely due to needing a degree of competency and flexibility in providing therapy, as well as the opportunities to be included in management and organisational decisions.

I was keen that it was the third‐year placement and ours is a third‐year placement because I suppose I see it very much as the leadership placement and fulfilling those competencies. 

[20]

#### 3.1.5 Orientation for Trainees is Indispensable

Given the differences between the traditional NHS placements that trainees may have previously experienced, staff felt that orientation to the service and the clients was essential before starting the placement.

We give people a very good induction and that's something I'm quite keen to do, so we make sure that people have a couple of weeks to really orientate themselves to working in the third sector. 

[20]

#### 3.1.6 The Value of Prior Learning on Homelessness and Reflective Practice

When considering how well‐prepared trainees were for their placements, staff discussed the importance of some prior learning about the context of homelessness and working in the third sector. Staff also thought it would be helpful if trainees were prepared to facilitate reflective practice.

Staff perceived that, for some trainees, the placement was the first time they had spent time with PEH and that they may have had to confront some of their preconceptions. Staff suggested trainees might be better prepared if they had some prior teaching or reflection on issues relating to homelessness. Additionally, reflective practice is considered very important for frontline staff given the complexity and trauma common for PEH. Some local authorities commission clinical psychologists to provide reflective practice and so the opportunity for trainees to facilitate reflective practice is considered a valuable skill to develop on placement and staff welcomed the chance to participate themselves.

[I] don't think people have had any teaching, usually on homelessness by the time they come here and that's in third year…and teaching in terms of consultation and reflective practice could always be helpful. 

[20]

### 3.2 Supervisor Perspective (See Figure 2)

**Figure 2:**
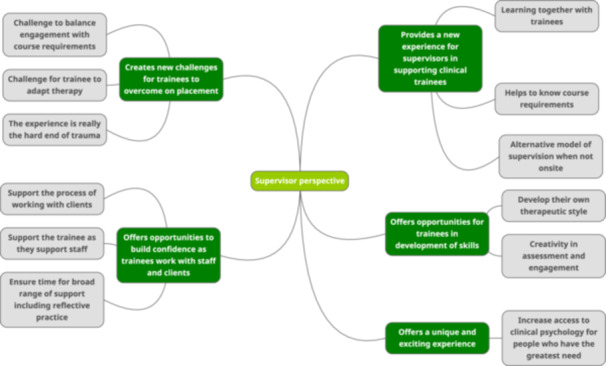
Thematic map for supervisor perspective.

Figure 2 shows the themes and subthemes resulting from the reflexive thematic analysis of supervisor interviews.

#### 3.2.1 Provides a New Experience for Supervisors in Supporting Clinical Trainees

Supervisors reported learning how to adapt therapy skills themselves, given the difference in settings from the NHS. They needed to explore models of supervision when not on‐site, for example, one off‐site visit or using role play, and how to adapt course requirements in the homelessness environment.

This has been quite a new set up in terms of how things operate in the third sector…I'm having to learn as I'm going along ‘cause I don't have that predated knowledge about how services work…the actual delivery of clinical supervision would be very similar, but I think the context and the background and the framework that I'm operating is very different. 

[18]

#### 3.2.2 Creates New Challenges for Trainees to Overcome on Placement

Given the prevalence of severe trauma, the flexibility of support that clients experiencing homelessness needed and the more chaotic nature of some services, it was challenging for trainees to adapt therapy with relatively little experience. Developing and consolidating therapeutic competencies is usually the starting point for placements. However, supervisors identified the placements as unorthodox, prompting trainees to adapt their therapeutic approach before they had learned the fundamentals of a particular model. Additionally, supervisors needed to help trainees understand the extent of trauma that many PEH were experiencing and the subsequent impact on attendance and engagement. This, in turn, presented a challenge to balance course requirements with the needs of staff and clients.

I think the experience is really, kind of, the hard end of trauma…it's reminding the trainee of that as well that once you can survive those DNAs [did not attend] and you learn not to personalise them, you see it as part of trauma and trust and intimacy and all the things that get disrupted developmentally when people have traumatic lives. 

[04]

#### 3.2.3 Offers New Opportunities for Trainees in the Development of Skills

Supervisors described how trainees could be creative in supporting assessment and engagement for clients, whilst developing their own therapeutic style. Whilst maintaining professional boundaries, trainees were able to adapt models of assessment and engagement and find creative solutions when working with PEH. Through the varied experiences and learning on placement, supervisors could see trainees develop their own identities and think about what kind of clinical psychologists they want to be.

A lot of really understanding where the resistance comes from. So somebody says, ‘Yes, I really want therapy,’ but then they don't turn up for the first session. The engagement process [has] been a very creative process to offer people lots of different ways of engagement. 

[04]

#### 3.2.4 Offers Opportunities to Build Confidence as Trainees Work With Staff and Clients

Supervisors could see how trainees built confidence over time, providing therapeutic interventions for clients and training and reflective practice for staff. The role of supervision was deemed to be crucial to provide a broad range of support to the trainee, guiding trainees as they put their learning into practice and supporting the well‐being of the trainees in this challenging environment. Trainees also developed assertiveness skills and established their authority in teams. Reflective practice was identified as particularly helpful.

There's the supervising in terms of supporting the trainees to develop their skills that they've learned at teaching through the uni and applying it to their population they're working with…and really important to acknowledge their experiences, their well‐being. 

[21]

So formal supervision, informal supervision, teamwork team meetings, in terms of modelling and encouraging the trainee to be involved in psychologically‐informed discussions within team meetings because otherwise it becomes very, how can I put it, very matter of fact, very one dimensional. 

[16]

#### 3.2.5 Offers a Unique and Exciting Experience

Supervisors felt that the homelessness placements offered a unique and exciting experience because of the flexibility and autonomy trainees were able to have in the services. There was the opportunity to be creative in supporting both clients and staff.

The nice thing in this placement is that there's that freedom and that creativity…you can create something and that's been quite exciting to have that freedom.

[04]

I think it's given them a completely new experience…I think the trainees can see that they're sort of innovators. They're able to be kind of guinea pigs and really develop things in the way that it was like, nothing's off the table. 

[18]

Supervisors were also excited about the opportunity for people with perhaps the greatest need, those experiencing homelessness, having greater access to psychological therapies.

It's very important that we recognise that the people with the greatest need, need the greatest input…[these placements are] an opportunity to reduce those health inequalities and improve access to psychological therapies. 

[16]

### 3.3 Trainee Perspective (See Figure 3)

**Figure 3:**
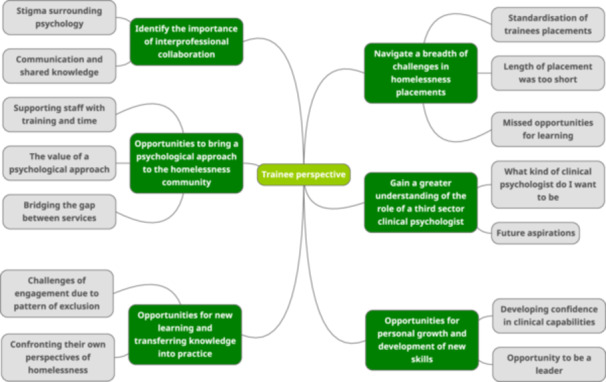
Thematic map for trainee perspective.

Figure 3 shows the themes and subthemes resulting from the reflexive thematic analysis of trainee interviews.

#### 3.3.1 Identify the Importance of Interprofessional Collaboration

Trainees believed that interprofessional collaboration, communication and sharing of knowledge between services helped to prevent delays to services and improve the overall care provided.

A focus at this placement has been bringing that psychological influence and into the sort of service delivery of others that I'm working with. 

[12]

However, collaboration has been proven to be more challenging in some services. Students felt there was stigma surrounding psychology and the role of trainee clinical psychologists, and that, without clear communication, some services may have viewed their presence as threatening leading to a reluctance to engage.

Maybe a preconception about psychology staff or what our role was going in, and maybe worried about being judged or saying the wrong thing to us. 

[10]

#### 3.3.2 Navigate the Breadth of Challenges in Homelessness Placements

Third sector homelessness placements come with a breadth of challenges. As homelessness placements are not configured like many NHS‐based placements, trainees often had to ask themselves how comfortable they were sitting with ‘uncertainty’. Trainees identified several challenges, including the importance of work‐based learning and the length and standardisation of placement. Trainees reported isolation and on occasion, missed opportunities for learning due to working across multiple services without a psychologist team.

Trainees also commented on the differences they experienced when starting the placement in homelessness and other placements in the NHS. Trainees felt they could have benefited from more structure and ‘clarity’ [06] to navigate their role and other services.

Maybe a little bit of structure in places. There was a lot of kind of self‐starting required. It was sort of like there are some things that you could do that could be helpful, but it's up to you and I think to start off with, I felt a little bit lost.

[11]

Placements are typically intertwined with the academic curriculum and course requirements; this relationship is less established in homelessness placements, with trainees explaining that it was ‘slightly harder to tick off some of the course mandated things’ [12] and trainees felt anxiety surrounding ‘how our course needs can be met on the placements’ [11].

Several trainees commented on the length of placement (i.e., 5 months), and while not exclusive to homelessness placements, some trainees felt that due to the nature of the placement, it was challenging to complete clinical work in the time frame and wondered if the placement length could have contributed to the low levels of client engagement.

If they're told, ‘Oh, you know, you're gonna be working with a trainee, she's gonna be there for five months.’ Whether that kind of puts them off straight away?

[13]

#### 3.3.3 Opportunities to Bring a Psychological Approach to the Homelessness Community

Psychological approaches to well‐being and practice are becoming more prominent in the homelessness sector. Clinical psychology trainees were able to expand the reach of psychologically informed interventions and approaches within homelessness services.

Trainees acknowledged the value of implementing a psychological approach in the homelessness community, noting its positive impact on placement staff and clients. For instance, staff well‐being is crucial in job retention, particularly in complex and high‐stress jobs often found in homelessness. Trainees reflected on the multifaceted role which placement staff have and the importance of supporting them.

You can't have a service that runs without staff, that the government so helpfully called unskilled labour, unskilled people because they are what makes the service work. You can't do this without them. So if they're unhappy, if they're finding things difficult, struggling to work with people, it's not going to work and the service is untenable, isn't it? 

[12]

National health services are often not set up to respond to the needs of PEH, with individuals placed on long wait lists or being excluded entirely. Trainees were able to bridge the current treatment gap between services by bringing psychological support into the services.

I'm seeing people who would otherwise slip through the nets of other mental health offers available. Our in‐house counselling service have like certain criteria and then obviously the NHS mental health services have their own criteria, and some people just don't quite fit either the inclusion criteria or like, won't quite do the right level of attendance….I'm filling a bit of a gap.

[03]

#### 3.3.4 Gain a Greater Understanding of the Role of a Third Sector Clinical Psychologist

Trainees were able to gain a greater understanding of the role of a third sector clinical psychologist; many were unaware of the role clinical psychologists could have in third sector services and homelessness.

This gave trainees the opportunity to ask themselves what kind of clinical psychologist they wanted to be. Unlike NHS‐based placements and services, trainees felt that working within homelessness gave them the opportunity to be creative when working systemically, as well as considering the prospect of continuing within this sector once qualified.

I do like that really systemic approach and kind of thinking about, you know, from lots of different angles how we can support people. So yeah, I do definitely think that's the kind of psychologist I want to be post qualification. 

[05]

#### 3.3.5 Opportunities for New Learning and Transferring Knowledge into Practice

Homelessness placements served as a platform for students to adapt theoretical knowledge into real‐world practice, as well as the opportunity to learn new skills.

Trainees acknowledged the challenges of engagement within this population, which was often attributed to a pattern of being let down. Trainees emphasised the importance of developing their therapeutic skills to effectively serve this population:

I think sort of developing your skills, learning to engage people was probably the most fundamental skill because the people who you are working with have been rejected by their families or by services or excluded from the society in many ways. 

[06]

Peer mentors were considered a ‘huge asset’ [08] to the trainees, with many accrediting peer mentors for their success in gaining individuals' trust and client engagement.

#### 3.3.6 Opportunities for Personal Growth and Development of New Skills

Trainees reflected on personal growth and new skills developed over the course of this placement. Unlike NHS organisations and placements, homelessness placements were viewed as more flexible, which was valued among trainees. This also allowed trainees to work outside usual psychological frameworks and interventions, instead develop their confidence in clinical capabilities and adapt their approach to meet the needs of their clients.

Likewise, trainees collectively acknowledged that homelessness placements provided an exceptional opportunity to cultivate leadership skills, distinct from any other placement experience.

In an NHS placement, I would go in and they'd have a case load of, you know, six people ready for you to see and you start working through that really. Whereas I think on a on the homelessness placement, you've got to create your own opportunities. 

[12]

### 3.4 Peer Mentor Perspective (See Figure 4)

**Figure 4:**
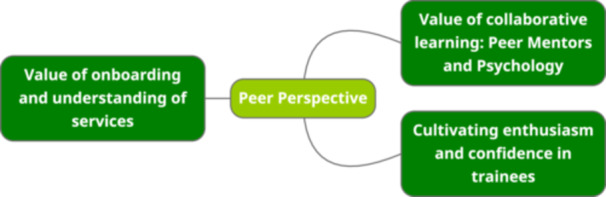
Thematic map for peer perspective.

Figure 4 shows the themes resulting from the reflexive thematic analysis of the peer mentor interview.

#### 3.4.1 Value of Onboarding and Understanding of Services

The peer mentor stressed the importance of a thorough induction process for trainees, helping them to transition into the role, given the complexity of the services and the clients they will serve.

“It's important that the trainees spend some time with the management of the services to really understand their business.”

The peer also thought an onboarding process would be a good opportunity for services to get to know the trainees. The emphasis on selecting the right trainee for a client could be a pivotal factor in achieving a meaningful therapeutic relationship and positive outcomes.

“One thing which I think is really important is the right trainee for client. It's not one‐size‐fits‐all. You have to be the right sort of person to fit with the client.”

#### 3.4.2 Value of Collaborative Learning: Peer Mentors and Psychology

Peer mentorship has the ability to foster a unique relationship, based on shared experiences, social support and role‐modelling recovery. Adopting a cooperative approach between peer mentorship and psychological approaches was considered instrumental for the well‐being of clients.

“I think the mixture of peer and psychology, is it a genius mix.”

“Just simplistic stuff like cognitive behaviour, coping mechanism skills, you can see that affecting the clients thinking and mentality.”

#### 3.4.3 Cultivating Enthusiasm and Confidence in Trainees

The peer mentor acknowledged the initial challenges experienced by some trainees during the placement's onset, as trainees appeared out of their depth, particularly if this sector was new to them. However, the peer observed a notable transformation as trainees began contributing positively to the client's well‐being, which led to heightened confidence and a growing enthusiasm for their roles and the homelessness sector.

“What I did see is the enthusiasm for our sector grow. Yeah, I know, I saw them really enjoy what they were doing, you know and making the difference as well.”

“When they first started they're a little bit unsure, being chucked in at the deep end and then they're confidence came. They tangibly saw difference they're making.”

## 4 Discussion

This study explored whether trainee clinical psychologist placements in homelessness settings could be a valuable pathway to improving provision and access to psychological support within homelessness services, both for clients and staff, and key to developing the workforce and a catalyst for the future recruitment of clinical psychologists in the third sector. The evaluation provided the perspectives of staff in homelessness services, supervisors of clinical psychology trainees, trainees and a peer mentor. Overall, trainee placements were beneficial for all parties and met the needs of trainee clinical psychologists as well as the services.

Staff saw placements as a way of getting previously unavailable psychological support into their services. They recognised that their perspectives on clients were changed positively, and clients were able to access much‐needed psychological support without long waits. Staff were grateful for the training and support, often lacking due to financial constraints. However, providing placements did provide a challenge as staff priorities are their clients and meeting the needs of trainee placement competencies sometimes felt at odds with client needs. For services, it met the needs of many clients and staff who would be unable to access support in a timely manner, reducing demand on NHS waiting times and fulfilling the Inclusion Health mandate [2]. Given the relatively short time frame of placements, and the challenges of engagement though, priority needs to be given to brief interventions that demonstrate efficacy. Evidence suggests brief motivational interviewing and CBT‐based therapies, including Acceptance and Commitment Therapy, can be effective in this population [21, 22]. In terms of staff support, trainees could be seen as merely filling a gap that will reopen after the placement but by trainees taking a psychologically informed approach to services, such as reflective practice, staff are equipped to continue this practice themselves once the placement has finished. Placements are instructed that trainees are supernumerary with limited time, but the risk of trainees being seen to plug a gap far outweighs the risk of not bringing clinical psychologists into the sector.

Supervisors identified placements as broadening the expertise of trainees in providing psychological support to PEH and homelessness staff, and a new experience in comparison to standard NHS placements; specifically, building resilience when navigating low engagement and skills to adapt conventional therapies. There was also the opportunity to build confidence and provide staff training and support in a challenging environment.

For trainees, it was an invaluable addition to their training in adapting treatments and developing their understanding of the population, which corresponds to the experiences of trainee emergency physicians in the United States serving PEH [14]. In line with trainee social workers' experiences of delivering psychological support to PEH on placements, trainees were concerned with being able to deliver appropriate interventions in the short time frame available [15]. However, for many, placements did provide an incentive to work in these settings once qualified due to overcoming anxieties and prejudices, and feeling better prepared for the environment, which mirrors experiences in mental health nursing placements [23, 24]). The unique environment of homelessness service placements facilitated the development of trainees by deepening their knowledge of professional boundaries, including flexibility and adaptability when working with traumatised clients; the value of a psychological approach in this client group, considering the effects of stigma; working alongside a peer mentor emphasising the importance of collaborative working with a wider range of colleagues; the need for flexibility and adaptability for both engagement and therapy; and providing staff training and support in this challenging environment. These opportunities developed trainees' confidence and competence, including building trainees' therapeutic style and leadership.

The peer mentor valued trainees because the need for psychological support is so great for PEH and so seeing the enthusiasm for working in this sector grow was exciting. Again, placements were identified as being challenging and requiring a thorough induction process.

The barriers to successful provision of placements in homelessness services encompassed engagement, professional expectations and access to clinical supervision, reflecting the literature on mental health placements [13]. Difficulties were associated with managing expectations regarding client attendance and engagement, attributed to the length of placement and challenges to complete clinical work in the time frame. Moreover, the disconnect between academic requirements and the realities of homelessness placements, for example, irregular engagement, added another layer of complexity. However, facilitators included providing the opportunity for interprofessional collaboration, highlighted by trainees as crucial for a successful placement. By working in a multidisciplinary setting, trainees learned how fostering collaboration between services to ensure integrated care was vital for preventing service delays and enhancing overall care quality [25].

Staff and the peer mentor emphasised how ensuring placements encompassed therapeutic techniques, societal issues and interagency collaboration enabled a multifaceted understanding of psychology within the third sector. Staff support when engagement posed challenges for individuals experiencing homelessness gave the trainees a unique opportunity to develop confidence in their clinical competence and find new ways to engage and work with clients. Likewise, clinical supervision for trainees as they adjusted therapy provided an opportunity for trainees to innovate in assisting clients and cultivate their unique therapeutic approach. In contrast to previous literature, trainees generally felt well‐supervised and had adequate support within these placements [13].

### 4.1 Strengths and Limitations

One strength of this study lies in the relatively large sample size recruited, especially considering the niche area and the recent emergence of DClinPsyc placements within homelessness services. Furthermore, the successful recruitment of individuals from various roles within the sector provided a diverse range of perspectives. However, a limitation was the recruitment of only one peer mentor, as having a broader range of perspectives from individuals with lived experience would have been advantageous.

Whilst member‐checking is considered the gold standard [26], the current study did not share transcripts or codes with participants due to time and availability constraints in this high‐pressure environment. The authors are exploring ways to reflexively collaborate with participants in future studies.

Whilst not completely mitigating potential response bias from participants who may be reticent to fully express their concerns due to the significant under‐resourcing of the sector and the desirability of placements providing extra support, participants were informed that the research team were independent of the DClinPsych programme and anonymity was assured.

### 4.2 Implications for Practice

Clinical psychology trainee placements could be key in the NHS long‐term plan to address health disparities and to improve access and outcomes for PEH outlined in the National Framework for Inclusion Health. The therapy offered by trainees removes the long waiting times for interventions and is flexible to cope with the unpredictable engagement of PEH. Trainees learn how to adapt therapy to meet clients' needs, benefitting both trainee and client, ultimately providing a substantial contribution to the long‐term improvement of Inclusion Health provision. Staff training and support contribute to the development of psychologically informed environments, arguably resulting in better outcomes for staff and clients. Due to the necessity for flexibility and adaptability, homelessness placements may be most appropriate for trainees with greater experience in their second or third year.

### 4.3 Implications for Future Research

This evaluation will lead to the improvement of clinical trainee placements in homelessness settings. Continued evaluation and application of learning from each cohort of trainees is needed to ensure that placements are developed and improved. Additionally, impact and outcomes for clients need investigating and reporting, ensuring placements are best serving PEH. Finally, measures of burnout and staff turnover could be gathered to explore these outcomes.

### 4.4 Conclusion

Clinical psychology trainee placements play a crucial role in advancing the NHS vision for Inclusion Health. These placements make a substantial contribution by helping prepare the health and social care workforce to establish sustainable provisions to improve the physical and mental health of PEH. This South East workforce development programme is presented as an example of good practice in the National Framework for Inclusion Health; this evaluation study has demonstrated the benefits from four different perspectives of those involved. The next steps are to evaluate the outcomes for those experiencing homelessness and collect longer‐term data on the impact of burnout and turnover on staff.

## Author Contributions

**Rebecca J. Ward:** writing–original draft, formal analysis, data curation, investigation, methodology, writing–review and editing, validation, software, project administration. **Frances T. Greenway:** writing–original draft, investigation, methodology, writing–review and editing, formal analysis, data curation, visualisation, software, resources. **Nick Maguire:** conceptualisation, writing–review and editing, supervision, funding acquisition, validation.

## Conflicts of Interest

The authors declare no conflicts of interest.

## Data Availability

The data that support the findings of this study are available from the corresponding author upon reasonable request.
